# When Emergency Patients Die by Suicide: The Experience of Prehospital Health Professionals

**DOI:** 10.3389/fpsyg.2020.02036

**Published:** 2020-08-28

**Authors:** Ines A. Rothes, Isabel C. Nogueira, Ana P. Coutinho da Silva, Margarida R. Henriques

**Affiliations:** ^1^Faculty of Psychology and Educational Sciences, University of Porto, Porto, Portugal; ^2^Center for Psychology, Faculty of Psychology and Educational Sciences, University of Porto, Porto, Portugal; ^3^Department of Clinical Nursing, Health Sciences Center, Federal University of Paraíba, João Pessoa, Brazil

**Keywords:** patient suicide, impact, prehospital emergency professionals, emotional reaction, narratives

## Abstract

The suicide of a patient can be a disturbing experience for health professionals. According to literature, a patient suicide is a professional hazard in the path of prehospital emergency professionals. In the emergency context, several factors pointed out in literature as predictors of increased emotional impact and more severe traumatic reactions are present. However, the impact of patient suicide on prehospital emergency professionals is still an understudied subject. The aim of this study was to better understand the impact and emotional reactions of prehospital emergency professionals facing a patient suicide, using a qualitative approach. Semi-structured interviews were conducted with 19 prehospital professionals. Fourteen narratives about a patient suicide experience were obtained. Three main categories emerged from the process of content analyses: (1) emotional impact and related factors; (2) perceptions of impact; (3) emergency context and professional growing. Death by suicide in the prehospital emergency context had a considerable emotional impact on these professionals. Several participants described intrusive thoughts and images as a consequence of attending to the death scene. Regarding the perception of impact, there seemed to exist a variation between the levels of *exposed to* and *affected by* suicide. There are specific features of the prehospital emergency context that emerged in the narratives of participants as factors which increased the patient suicide impact, namely attending the death scene, encountering the family or other survivors, and managing the feeling of responsibility for not arriving in time of the rescue. The narratives of prehospital professionals also indicated some negative effects on their professional practice, such as doubts about their competence, training, and limits to liability. The death of a patient by suicide in the prehospital emergency context can be a difficult experience, marked with an intense emotional impact. Nevertheless, it can have some positive effects, such as professional growth and increased awareness for the phenomenon. Specific training appears to be fundamental to promote professional growth and to overcome the negative emotional impact.

## Introduction

A significant number of prehospital emergency professionals is likely to have experienced a suicide case in their practice. Indeed, the suicide of a patient is a professional hazard in the emergency context (Gaffney et al., 2009). A persons’ suicide is a very emotional issue and it is expected to have emotional effects on others. The exposure to death by suicide may cause psychological, physical, and social suffering even if the deceased is unknown (Andriessen and Krysinska, 2012; Cerel et al., 2014). Despite its relatively frequent nature and the specific context of the emergency help, which generally implies a brief contact and a limited relational closeness, a patient suicide impact should not be undervalued.

The impact of a patient suicide has been studied in different groups of health and mental health professionals, including psychiatrists (e.g., Chemtob et al., 1988a; Courtenay and Stephens, 2001; Henry et al., 2004; Ruskin et al., 2004; Rothes et al., 2013b; Castelli Dransart et al., 2014), psychologists (e.g., Gorkin, 1985; Chemtob et al., 1988b; Kleespies et al., 1993), physicians (e.g., Halligan and Corcoran, 2001; Kendall and Wiles, 2010; Rothes et al., 2013a), nurses (e.g., Akeshi et al., 2003; Takahashi et al., 2011; Castelli Dransart et al., 2014), and social workers (e.g., Jacobson et al., 2004; Castelli Dransart et al., 2014). However, to the best of the authors’ knowledge, only four studies (Gaffney et al., 2009; Nilsson et al., 2017; Vedana et al., 2017; Nelson et al., 2020) approached the theme of suicide impact in prehospital emergency context. These studies revealed important data, but the subject remains understudied. The delivery of prehospital care varies greatly between countries (e.g., Fairhurst, 2005), limiting transferability of results to dissimilar settings. Gaffney et al.’s (2009) study used a multidisciplinary sample, including emergency medical technicians. The authors found that emergency technicians, along with psychiatrists, were the groups with the highest incidence of patient suicide and highlighted the need for further research to better understand the specificities of the context related to this experience (Gaffney et al., 2009). Nilsson et al. (2017) conducted a qualitative study about the support provided by prehospital emergency professionals to the survivors. This study showed specificities of the prehospital emergency context, such as the feelings of inadequacy and uncertainty about the professional responsibility over the situation (Nilsson et al., 2017). Prehospital emergency professionals are usually the first health professionals that survivors contact with and even though they are strangers to each other, that temporary crisis may lead to an experience of shared closeness. According to the author, emergency professionals tend to range between an ethical conflict and compassionate reactions toward the survivors (Nilsson et al., 2017). The study of Nilsson et al. (2017) was focused on support and responsibility of prehospital professionals toward survivors, not addressing the emotional impact of patient suicide *per se*. The study about the experience of nurses in assisting people with suicidal behavior in prehospital context conducted by Vedana et al. (2017) reinforces that this assistance is a demanding and even disturbing experience in the emergency work. Although Vedana et al.’s (2017) study also includes the experience and impact of death by suicide, it is not always clear when it is referring to suicides or suicide attempts. A very recent study (Nelson et al., 2020) highlighted that ambulance staff presented long-term and emotionally impacting memories of suicide cases and often had difficulties to deal with the persons bereaved by suicide at the emergency setting, reporting uncertainty, anxiety, and lack of training. Nelson’s study used a small sample of paramedics and technicians and was conducted in the scope of a prehospital emergency setting based on the Anglo-Saxon model, centered essentially on the activity of paramedics, “of taking the patient to the hospital” (Dick, 2003; Al-Shaqsi, 2010). Thus, this limits transferability of results to contexts that adopt the Franco-German model of “taking the hospital to the patient,” as is widely implemented in Europe (e.g., Al-Shaqsi, 2010).

There is a general agreement in the relevant literature that a patients’ suicide can be a disturbing experience for health professionals (e.g., Rothes et al., 2013a, b, 2017; Séguin et al., 2014; Castelli Dransart et al., 2017) and even trigger traumatic reactions (Chemtob et al., 1988a, b, Cryan et al., 1995; Gaffney et al., 2009; Castelli Dransart et al., 2014). Despite the fact that losing a patient to suicide generates stress and emotional suffering among health professionals, these emotions rarely present clinical levels of significance (Séguin et al., 2014). Furthermore, this event could be an opportunity for professional growth and to increase skills to manage suicidal patients (Rothes et al., 2013a, b).

Specific factors have been identified as predictors of an increased emotional impact and more severe traumatic reactions (Castelli Dransart et al., 2014, 2017). These factors are related with the characteristics of the context of prehospital emergency work, such as the attendance at the death scene (Gutin et al., 2011; Cerel et al., 2014) and the feeling of responsibility (Castelli Dransart et al., 2017), namely for saving life. In general, health professionals guide their practices and decision-making based on the moral and ethical principle that one should always try to save a person’s life, and the extent of this responsibility depends on the context of intervention (Mishara and Weisstub, 2005). Indeed, prehospital emergency professionals work to assure an effective and rapid health caregiving to the victims (Instituto Nacional de Emergência Médica, 2019). In this context, the professionals’ main obligations are to provide emergency care at the setting of the occurrence, to provide an assisted and safe transportation of the patient into the hospital, and to provide adequate information to guarantee the coordination between all care systems involved (Instituto Nacional de Emergência Médica, 2019). The National Institute of Medical Emergency (INEM) follows the denominated Franco-German model of emergency care delivery, which is based on the “stay and stabilize” philosophy (e.g., Al-Shaqsi, 2010). At the INEM, around 80% of human resources are frontline professionals: 356 physicians, 266 nurses, and 756 prehospital emergency technicians, who are available for direct contact with all the incoming emergency calls (Instituto Nacional de Emergência Médica, 2018). In Portugal, in the year 2018, a total of 1,319,443 emergency activations were registered, of which 32,251 were directly related to psychiatric or suicidal problems (Instituto Nacional de Estatística, 2018).

Despite its evident importance, there is a paucity of literature about how prehospital professionals emotionally deal with patient suicide focusing on their experiences. To improve postvention support in this context, there is a need to better understand the experiences of the prehospital emergency professionals when a patient dies by suicide.

As key to suicide research and prevention activities in recent decades, the importance of postvention and those with lived experience was acknowledged (O’Connor and Portzky, 2018), in which it may be relevant to include the experience of health professionals. Therefore, the aims of this study were to understand the impact and emotional reactions of prehospital emergency professionals facing a patient suicide and to explore the existence of specificities related to the prehospital emergency context.

## Materials and Methods

The present study is part of a larger qualitative investigation that aimed to explore the conceptions, practices, and experiences of prehospital emergency professionals toward suicidal patients. The data used in the present study focused on the theme of professionals’ experiences, narrated by the participant during the interview. At that moment, professionals were invited to recall and report an impactful suicide attempt attendance with as much detail as possible, recollecting the whole process of care, their attitudes, feelings, and difficulties. Most of the professionals described cases of death by suicide indicating the interest of this study’s focus on patient suicide impact on prehospital emergency professionals.

### Sampling Process and Participants

The participants of this study were prehospital emergency professionals from the INEM, specifically professionals who work at the front line – physicians, nurses, and prehospital emergency technicians.

A combined methodology was used in the sampling process: (1) convenience, (2) snowball, and (3) emergent subgroup. (1) Convenience sampling started with few information-rich participants from each catchment region, who were easily accessible to the researchers and (2) snowball sampling, asking each participant for contacts of others who could also provide their perspectives, creating a chain of participants. (3) Furthermore, during fieldwork researchers realized that a significant number of participants described cases of death by suicide rather than suicide attempt, as was asked and while others described both situations. That is, patient suicide impact in the prehospital emergency context emerged as a critical issue and this emergent subgroup of prehospital professionals who described patient suicide situations became an information-rich sample providing a specific insight into the understudied phenomenon of patient suicide impact on prehospital professionals. Thereby, during data collection, the pertinence of studying patient suicide impact in prehospital emergency professionals arose. Furthermore, it was planned in advance to pursue the three following criteria: (a) homogeneity, (b) heterogeneity, and (c) saturation. (a) Homogeneous sampling would be applied regarding the specific experience of suicidal cases (participants must have experience with suicidal patients in the prehospital emergency context) and (b) heterogeneous sampling would be followed regarding professional group (physicians, nurses, emergency technicians), years of experience, and also region of the country. Two different catchment regions were selected based on their different characteristics. One was a large urban center that also covers suburban areas with high density, located in the North of the country, and the other one was a region with less density, including rural areas and located in the Center of the country. (c) Another planned criterion for sampling was the saturation or redundancy of collected information, that is, the strategy was to stop the chain of recruitment of participants when no new information was forthcoming from the new interviews. Based on methodological recommendations (Guest et al., 2006), it was estimated that about 12 interviews would be necessary to reach saturation.

From the 19 professionals composing the global investigation, 14 described cases of death by suicide as the most impactful experience in this specific practice area. Among these participants were seven women and seven men. Regarding their occupation, there were four physicians, eight nurses, and two prehospital technicians. Participants’ ages ranged between 34 and 55 years (*M* = 41.6; SD = 5.8). Years of professional experience in the health sector varied from 8 to 33 years (*M* = 17.1; SD = 7.4) and the years of experience at INEM varied from 4 to 19 years (*M* = 10.6; SD = 5.7).

### Data Collection

Data were collected by means of a semi-structured interview organized into three main themes: conceptions about people who attempt suicide, prehospital practices used in cases of suicide attempt, and professionals’ experiences in this area. The topics of the interview were based on conclusions of previous researches (Rees et al., 2014, 2015, 2017). Moreover, a small questionnaire was used to collect socio-demographic and professional data, including information about training on suicide prevention. The data present in this study were followed by the next guiding question: “I would like you to think about the suicide attempts you attended in this service so far. Among them, can you select one that has particularly affected you or that has been more significant for you? Furthermore, I would like to ask you to describe the situation and your own experience, in as much detail as possible.”

Participants were personally contacted and invited to voluntarily participate in the study. All of them signed an informed consent approving the audio recording of the interview and the use of data for research purposes guaranteeing confidentiality of their personal information. Most interviews were conducted in the participants’ workplace and lasted approximately 35 min (range: 15–60 min).

### Data Analysis

All the interviews were fully transcribed, and data were submitted to content analyses following the methodological recommendations of Bardin (1977) and in a more systematic and detailed way, following the thematic analysis as formulated by Braun and Clarke (2006, 2013). Inductive thematic analysis was used to generate an analysis from the data (from the bottom up) (Braun and Clarke, 2006, 2013). To analyze the data, no *a priori* coding categories were identified, and themes were allowed to emerge from the data. However, and as exposed by qualitative authors (e.g., Braun and Clarke, 2013), despite categories not emerging from theory, the analysis was also shaped by the standpoint of researchers and their disciplinary knowledge. Based on Braun and Clarke (2006, 2013), the researchers conducted the systematic analysis through a six-step process: (1) First, the researchers became familiar with the data by reading each transcript twice – one first reading in a fluid way and in a second reading, during which the initial ideas for coding were written in the margins of files. (2) In the second step, the initial codes were generated by two researchers (the two first authors), who systematically coded each unit of meaning across the entire data set and collated data relevant to each code. To identify aspects of the data that relate to research objectives, researchers did a complete coding across the entire dataset. To code, a manual hard-copy process was used and both kinds of codes were used: semantic and latent. That is, mainly codes were used that relate the semantic content of the data (data-derived or semantic codes) but also researchers-derived or latent codes. Researchers who coded the transcribed interviews worked through each interview in full, before proceeding to the next. (3) The third phase entailed sorting the codes into potential themes. In these second and third phases, the two researchers – first and second author – worked together to reach consensus. According to guidelines for qualitative research (e.g., Elliott et al., 1999), two researchers were used as analysts (first and second authors) and two researchers (third and fourth author) for a verification step. (4) In the fourth phase, themes were reviewed and refined, checking whether the data cohered together meaningfully within each theme. (5) In the fifth phase, final themes were defined and (re)named. (6) In the sixth phase, the report was written, and excerpts from participants’ interviews were selected to illustrate each theme. In these fifth and sixth phases, the third and fourth authors reviewed data to confirm and expand findings (Hill, 2012).

## Ethical Considerations

The study protocol was approved by the Ethics Committee of the Faculty of Psychology and Educational Sciences of University of Porto (Ref^a^: 2019/02-1).

Anonymity and confidentiality of the participants was guaranteed throughout the study, according to the Portuguese law of data protection.

## Results

**FIGURE 1:**
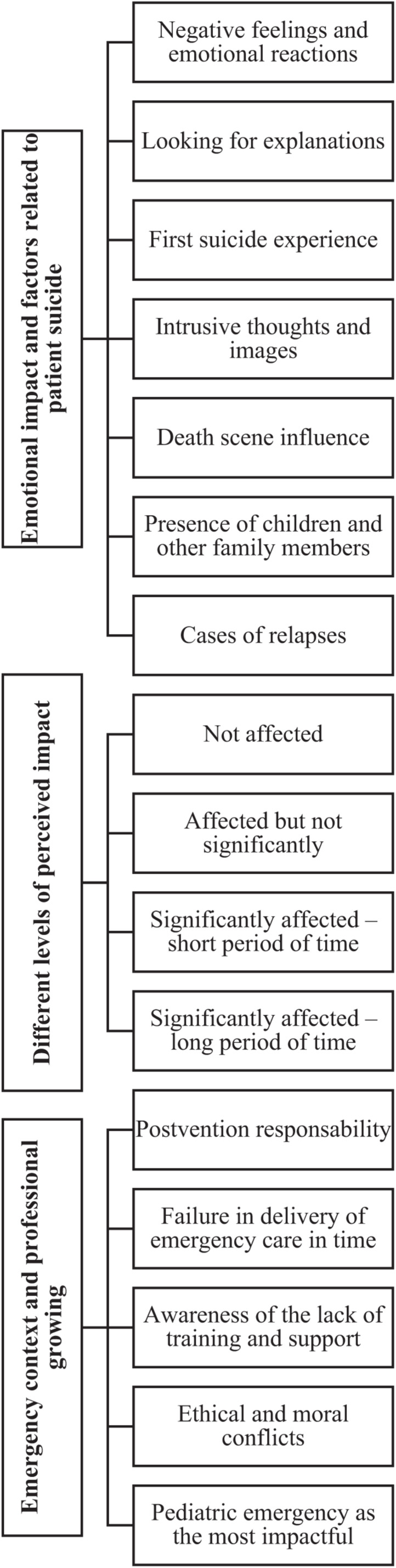
Summary of categories from the prehospital professionals’ interviews content analysis.

Content analysis resulted in 3 main categories and 15 subcategories conveying the prehospital professionals’ patient suicide experience. Findings are summarized in Figure 1.

### Emotional Impact and Factors Related to Patient Suicide Impact

#### Negative Feelings and Emotional Reactions

Prehospital professionals reported a set of feelings and emotional reactions revealing that a death by suicide in the emergency context is likely to have a considerable emotional impact on the health professionals. Overall, it was difficult for these professionals to identify a specific emotion felt toward a suicide situation. Instead, professionals reported mixed feelings as anger at the deceased, guilt for not arriving on time, feeling sorry for the families, incomprehension, horror in shocking situations and, in few cases, empathy.

*“And the fact that I knew that the last time I arrived on time*… *and this time I didn’t*… *it’s complicated, it’s complicated to digest*…*”* (Participant 10).

*“That context had a particular impact on me, perhaps because it wasn’t difficult for me to relate to that man.”* (Participant 6).

#### Looking for Explanations

The emergency professionals often revealed an attempt to understand why the person died by suicide:

*“Probably because of some family problem, maybe a divorce, he isolated himself in that hotel room. (*…*) I think in that moment he felt cornered and ended up killing himself.”* (Participant 6).

*“He lived alone, and his wife had just died. I think it was all this anguish that lead him to suicide.”* (Participant 10).

#### First Suicide Experience

The first suicide attendance experience seems to have a particular impact on the prehospital professionals, emerging as the most impactful case for some of them:

*“The one that affected me the most was perhaps the first hanging, because I’d never seen one before.”* (Participant 1).

*“I had just started my work in the emergency service. I think it was my first suicide* [attendance], *at least the first I can recall, and it was a far-fetched situation.”* (Participant 2).

#### Intrusive Thoughts and Images

Different participants described intrusive thoughts and images as consequence of attendance to the death scene, revealing that a patient suicide may trigger stress and traumatic reactions.

*“Despite my job not giving me nightmares, until today I could still evoke the clothes he was wearing, the way he was hanging, describe everything upside down*…*”* (Participant 4).

*“This is the situation I remember the most and I think it will stay in my mind for many years.”* (Participant 5).

*“It was the situation in which I had more flashes in my head. That is to say, I went to sleep, I woke up and that would still come to my mind. It was terrifying.”* (Participant 13).

#### Death Scene Influence

The prehospital emergency health professionals described the death scene as an aspect that impacted and affected them. Some of them reported symptoms of stress and traumatic reactions, such as intrusive thoughts and images, feelings of shock and horror, and sporadic insomnia related to the suicide context and its characteristics.

In this study, the death scenario appears as a factor related to a greater impact in different situations including beautiful scenarios in which the shock comes from the contrast between the fatality and the beauty of the outdoor space:

*“The view was so beautiful, and it was a very sunny day. It was a place where no one expected something bad could happen. I remember arriving to the scene and thinking to myself ‘How could someone commit suicide here?”* (Participant 2).

Horror situation, mainly if the body was deformed or mutilated:

*“I arrived at the scene and found a 36-year-old young lady floating in 5 liters of blood in bed. She was found like this by her 17-year-old son. This was a Dantesque scenario.”* (Participant 5).

*“It was terrifying, a terror, a complete beheading*… *During months, it kept coming back to my mind all the time.”* (Participant 13).

And situations in which the scenario reveals the anticipation and detailed planning of everything:

*“What struck me was the plan he had put together before he did that (*…*) That table was impressive, everything in detail, that felt-tip pen*… *Whoever got there would know exactly what to do.”* (Participant 13).

For some participants, having contact with specific details was very impactful:

*“In this case, it was the detail. Everything was so tidy, the dogs were missing, the gun which wasn’t his but from his notary friend. The detail of the registers, the bills yet to be paid and instructions on how the family should proceed to pay them off. Also, the letters all set to be sent to the family members, with the posting money aside.”* (Participant 7).

*“At first, I thought it was only one more suicide, but then what had the greatest impact on me was a farewell letter he had with him. Later, in the emergency room, we were able to read it and his words really affected me.”* (Participant 14).

In this last case, reading the farewell letter may also have meant an emotional approach to the deceased.

#### Presence of Children and Other Family Members

The presence of children or other vulnerable relatives in the emergency situation was described as a factor of increasing stress and difficulties.

*“The difficulty in that situation was not with the patient itself, who was already dead, but with the wife and the children standing there, looking at us with so many expectations about our work.”* (Participant 11).

*“That situation affected me. We couldn’t do anything to the victim because she was already death at our arrival. But that little girl, probably around eleven years old, she was clearly disturbed. In the first moment, she didn’t even want to talk to us*…*”* (Participant 12).

Regarding the presence of children or other survivors in the suicide scene, some prehospital emergency professionals describe an emotional alienation as coping strategy and a mechanism of self-protection.

*“At some point, we create compensation mechanisms. Mechanisms that lead us to isolate ourselves, to push us a little away from some things. At some point, I think that’s what we do. (*…*) I tell myself ‘I have to block this, this is not mine.’ Even to protect ourselves.”* (Participant 7).

*“*… *then I must go away. It is work. I get there, I take care of it and I go away.”* (Participant 4).

#### Cases of Relapses

When the patients were previously attended by the professional, the death by suicide seems to have had a greater disturbing effect and was described associated to feelings of guilt and frustration. Specifically, they reported guilty because the first attempt “*I arrived on time*… *and this one I didn’t arrive in time”* (Participant 10).

### Different Levels of Perceived Impact

The professionals’ narratives revealed the existence of different levels of effects of being exposed to the patient’s death by suicide:

#### Not Affected

*“I clearly admit that I see a suicide attempt as a situation of normal pathology. It does not cause me any inconvenience or any*… *How can I say*… *any additional emotional impact.”* (Participant 12).

#### Affected but Not Significantly

*“There was a situation that affected me. A grandfather was accused of abusing his granddaughter. So, he wrote a letter saying that he had never done anything to the girl and that he was tired of being accused by everyone. But if it was true or not, we’ll never know.”* (Participant 3).

*“The impact has to do with the way you perceive what you see when you get there. In my case I don’t recall any situation that had greater repercussions nor that kept me thinking about it much longer.”* (Participant 1).

#### Significantly Affected – Short Period of Time

*“I went to sleep, I woke up and that would still come to my mind, or during my daily routine. It was terrifying, a terror, a complete beheading*… *During months, it kept coming back to my mind.”* (Participant 13).

#### Significantly Affected – Long Period of Time

*“That is the situation that I remember the most and I think I’ll remember it for years (*…*) She was lying in a pool of blood, her skin was thin and pallid like paper.”* (Participant 5).

Some professionals identified themselves personally with the case, revealing an emotional entanglement with the situation and perhaps a countertransference mechanism, contributing to a great emotional impact and typical grief reactions.

*“It’s hard to deal with. It’s because*… *my husband’s grandfather also committed suicide*… *these are always situations that mark us because they remind us of a lot of old things.”* (Participant 10).

### Emergency Context and Professional Growing

#### Postvention Responsibility

Prehospital emergency professional’s revealed specific concerns regarding family, and especially toward children and adolescents. This is one of the reasons that trigger psychologists’ presence to be requested at the death scene.

When faced the survivors, some prehospital professionals revealed a high sensibility beyond their sense of responsibility:

*“No matter how short the time we have in there is, the few words we say to the family can be very important and impact them forever, in a positive or negative way*… *In this traumatic moment, we are very important for those people.”* (Participant 11).

Other reported that their responsibility is to activate the professional assistance of a psychologist at the death scene and that they should only focus on the tasks they were trained to do and quickly get out of there.

#### Failure in Delivery of Emergency Care in Time

Suicide cases placed prehospital emergency professionals in the face of incapacity, challenging their competence and mission to save patients, keep them alive, and take them safely to the hospital. For some participants, the suicide was interpreted as a failure to rescue, namely a delayed emergency help – “*we did not arrive in time*.”

#### Awareness of the Lack of Training and Support

Faced with the difficult challenge of dealing with family members or other survivors, many prehospital professionals realized the insufficient education and training they have. These professionals stated that it would be useful to receive postvention training to promote professional growth in this area.

*“I have no training in giving bad news, I have no training in dealing with a suicide attempt, I don’t have it.”* (Participant 2).

*“No one teaches us in college how to give bad news. We learn it from experience and those who have interest go and search for it*…*”* (Participant 7).

*“I openly assume I am not fully trained or able to talk with the patient or the family. That is the hardest part*… *There should be more training, there should be more support.”* (Participant 4).

The professionals’ narratives also indicated some effects on professional practice, including self-doubt and questioning of the limits of responsibility, awareness about the lack of training on suicide prevention and the need to use emotional strategies of self-protection.

“*That’s the hardest part*… *There should be more training, there should be more support [*…*] He was about twenty years, it was by hanging, we have to inform the parents, it was horrible, it was very complicated.”* (Participant 4).

The lack or the insufficient formal help system in these cases, regarding survivors and professionals, also arose from the narratives of prehospital emergency professionals.

#### Ethical and Moral Conflicts

Some narratives revealed that situations of suicide give rise to ethical and moral conflicts. Suicide in the context of prehospital emergency seems to put professionals in a confrontation between restricting their work to the basic goal of saving lives, focusing on issues of physical stabilization, and feelings of ethical responsibility and moral sensibility toward survivors. There is also a conflict between the sensibility and willingness to protect survivors and the need to protect themselves, thus facing the limits of their moral responsibility and their professional limitations.

#### Pediatric Emergency as the Most Impactful

Different prehospital professionals explained that the most impactful emergencies were pediatric situations rather than suicidal behaviors:

*“In my opinion, there are more traumatic situations than suicide attempts. I think it may depend on the circumstances. For example, if children were involved in the situation, even just as bystanders, it would gain another dimension.”* (Participant 2).

*“Now I am thick-skinned. Whatever comes, it doesn’t interfere that much with me anymore. But, if they are children, it still upsets me a lot.”* (Participant 13).

## Discussion

In the authors’ best knowledge, this is the first available study to investigate specific distressing experiences about a patient suicide in the prehospital emergency context, covering nurses, physicians, and prehospital emergency technicians. Thus, it brings important findings to postvention, particularly regarding the impact on prehospital health professionals. The emerged categories show similar impact characteristics to those found in previous studies with other professional groups (e.g., Rothes et al., 2013a, b; Castelli Dransart et al., 2014), as well as highlight some specificities of the emergency context, which may increase emotional impact and traumatic reactions. The present study adds data to the few previous studies, which approached the experience of suicide cases in prehospital emergency context (Gaffney et al., 2009; Nilsson et al., 2017; Vedana et al., 2017; Nelson et al., 2020).

The fact that death by suicide is one among many causes of death or trauma to which emergency professionals can be exposed to may explain that patient suicide impact on emergency professionals has been less studied than in other professional groups.

The narratives of the participants in this study add further evidence that a patient suicide can be a disturbing experience for the health professionals involved, demanding adequate postvention measures to re-establish the well-being of the professional. To date, this was already expectable when considering psychologists, psychiatrists, GPs, nurses, and social workers (e.g., Castelli Dransart et al., 2014, 2017; Séguin et al., 2014), but the present study adds original empirical data that reinforce the importance of not neglecting the effects of suicide on the prehospital emergency team, which in turn can be fundamental to rescue people at risk and even influence the future therapeutic process. The provided care by prehospital emergency professionals is fundamental because it can influence future decisions of help-seeking (Royal College of Psychiatry [RCP], 2010). In this study, prehospital professionals reveal personal resources to functionally cope with the suicide case, as found in previous studies (e.g., Pieters et al., 2003; Rothes et al., 2013b); however, it is not negligible that there are descriptions of increased difficulties in the aftermath of the event, such as stress reactions and insomnias, which may indicate being in need of help.

In this study, prehospital professionals looked for possible reasons that led to suicide and reported them in their interviews, revealing the need for reintegration of the suicide experience as a loss that needs to be taken into account. This search for answers is a normative process of adaptation after suicide (e.g., Postuvan, 2017) and it occurred even when prehospital professionals did not know the deceased. This process is part of the human nature and entails a social role in the stability and assurance of social values (Palgi and Abramovitch, 1984).

Among the experiences described in this study, professionals pointed several reasons for their choice of the traumatic suicide event. Often the experience chosen as the one which caused more concern and anguish was the first time a professional saw a suicide in the emergency context. Thus, the results of this study somehow reinforce the findings of previous studies, which showed that having less experience may increase the emotional impact of the death by suicide of a patient (Chemtob et al., 1988a; Wurst et al., 2010). However, there is no consensus in literature regarding the influence of years of experience on the impact of a patient suicide, as different studies achieved different results. Findings from Chemtob et al. (1988a, b) studies showed a decreasing impact with the increasing of age and years of practice among psychiatrists, whereas in a similar survey with psychologists there were no significant differences. Gulfi et al. (2010) found that young professionals have a tendency to make greater changes in their practice than older colleagues but found no differences regarding the intensity of emotional impact. Other two studies concluded that the intensity of reactions and distress in the aftermath of a patient suicide was independent from age and years of experience (Hendin et al., 2000; Hendin et al., 2004).

As also found in previous studies with different professional groups (e.g., Chemtob et al., 1988a, b; Cryan et al., 1995; Gaffney et al., 2009), some prehospital professionals reported that the most impactful patient suicide triggered traumatic symptoms, such as intrusive thoughts and images. Furthermore, these findings support the conclusion that the greatest impact is more likely to occur when several risk factors are involved and associated to the lack of support and training (Castelli Dransart et al., 2017). Indeed, different predictors of stress and traumatic impact defined in literature were described by those participants who had stress reactions in the aftermath of the death by suicide of an emergency patient, namely, a shocking death scenario (Gutin et al., 2011), the lack of training and support (Castelli Dransart et al., 2017), and a closeness with the patient (Cerel et al., 2014, 2017), even if it is a subjective closeness, through identification or countertransference mechanisms (e.g., “*It was a person of my age*”). Nelson et al. (2020) also found this mechanism of identification as a potential risk factor for a greater distress. In agreement with findings of the influence of both the emotional closeness and the exposure to a higher number of suicides as potential factors that increase negative consequences (Van Orden et al., 2010; Cerel et al., 2017), in this study the rescue to relapses seems to have had a greater disturbing effect. This situation of the suicidal behavior’s repetition emerged associated to a specific category of the emergency work context – the feeling of responsibility for not arriving in time. The responsibility for the care was identified in previous studies as a predictor of an increased emotional impact (Castelli Dransart et al., 2017).

This study reinforces the conclusion that the death scene may affect the emotional impact but adds that not only a horror scenario or a mutilated body may be predictors of increased negative impact, as identified in previous studies (e.g., Gutin et al., 2011; Nilsson et al., 2017). The reactions and negative feelings may also be more intense if there is a contrast between the tragic event and the context or if the professionals had contact with specific details, such as a suicidal farewell letter. This in turn may increase the perceived closeness with the deceased acting as moderator of the impact, as already pointed out in the literature (Cerel et al., 2014, 2017; Castelli Dransart et al., 2017).

Prehospital professionals revealed feelings of responsibility toward the survivors, concerns about family needs and protection from the intense situation, especially when children are involved. They also reported doubts, feelings of uncertainty and incapacity and lack of skills to deal with the survivors, which sometimes led to contact the psychological emergency staff. These feelings and doubts regarding the care of survivors are in line with the outcomes of Nilsson et al. (2017) that studied the experiences of facing the family of a deceased by suicide in the prehospital emergency context. These findings are also in accordance with Nelson et al.’s (2020) study, which concluded that having to deal with the intense emotional reactions of bereaved individuals is a difficulty for the paramedics. The loss of a patient by suicide and the coping processes of the prehospital emergency professionals of this study varied from professionals whose exposure to suicide did not appear to affect them (although it may be a process of denial as a defense mechanism) to professionals whose narratives included normal grief reactions, even though they did not last long. Adopting the categories of terminology proposed by Cerel et al. (2014), this study found that the perception of impact apparently varies between the levels of exposed to affected by suicide; the levels of bereaved were not found. These results are consistent with those found by Séguin et al. (2014). The authors reviewed 37 studies about the impact of a patient suicide and concluded that for the majority of health professionals the loss of a patient by suicide may often trigger grief reactions, although they are not part of a grieving process, namely because professionals recovered fast. Moreover, the findings of this study showed that the death by suicide may have a constructive consequence on the prehospital emergency professionals, increasing the awareness of the importance of training and support in this field. Several participants pointed out the lack of preparation and training as a difficulty. The interviews created an opportunity for professionals to reflect and increase their awareness about the suicide phenomenon. Previous studies had already highlighted the potential constructive effect that patient suicide may have in the professional growing (e.g., Rothes et al., 2013b), especially if proper training and support is provided. Thus, the present study underlines that providing more training on suicide prevention for those working in prehospital emergency context is fundamental. This specific education should include the anticipation and preparation for patient suicide. Moreover, the training aimed at prehospital professionals should approach specific features of the emergency context, mainly those that participants of this study highlighted as risk factors for increased negative impact and stress reactions: cases of relapses, death scenario, presence of children and other survivors, details’ knowledge and emotional closeness, and not to arrive on time.

Future research on suicidal behaviors in prehospital emergency context should explore the relationship between the professionals’ conceptions toward suicide and patient suicide impact and study the effects of socio-professional variables, including training and years of experience in prehospital emergency care. Given the wide range of time intervals between the case chosen to be described as the most impactful and the moment of the interview (among the professionals who referred this aspect), it is advisable to systematize and analyze this variable in future studies. Future research should investigate the experience of psychologists who have experienced patient suicide in the specific prehospital emergency context.

### Limitations

The study only covers two regions of the country. Besides the fact that suicide presents different characteristics depending on the area of the country, social and cultural variability inter regions may have influenced the way prehospital emergency professionals deal with the patient suicide experience. Another limitation is that interviews were conducted within the context of the professionals’ work and in some cases, it had to be interrupted and later restarted, losing some depth and fluidity.

## Conclusion

In general, a suicide case in prehospital context seems to be an impactful experience for the professionals, triggering traumatic and stress reactions for some. Further research on contextual variables and about the relation between perceived impact and coping strategies can provide useful clues to be integrated in formal training in suicide and eventual postvention measures aimed at prehospital emergency professionals.

## Data Availability Statement

The datasets generated for this study are available on request to the corresponding author.

## Ethics Statement

The study protocol was approved by the Ethics Committee of the Faculty of Psychology and Educational Sciences of University of Porto. The participants provided their written informed consent to participate in this study.

## Author Contributions

IR contributed to the conception of the research and study design, participated in the data collection, analyzed and interpreted the data, planned and wrote the draft of the manuscript in full, and wrote and rewrote large sections. IN contributed to the conception of the research and study design, collected most of the data and transcribed the data, analyzed and interpreted the data, and wrote parts of the manuscript. AC contributed to the conception of the research and study design, participated in the collection data and transcription, contributed in the data analysis (as reviewer), contributed in the discussion and interpretation of data and in references search, and provided some critical clues. MH contributed to the conception of the research and study design, contributed in data analysis (as reviewer), questioned and discussed the outcomes and interpretation of the data, and supervised the work in all stages. All authors contributed to the article and approved the submitted version.

## Conflict of Interest

The authors declare that the research was conducted in the absence of any commercial or financial relationships that could be construed as a potential conflict of interest.
